# Vitamins C and E alleviate the deleterious effects of electronic cigarettes on tongue muscles and nerves in rat model

**DOI:** 10.1186/s12903-025-06523-z

**Published:** 2025-07-16

**Authors:** Yasmine M. Tolba, Raghda A. Abou ayana, Dina A. Nagui

**Affiliations:** 1 Lecturer of Oral Biology, Faculty of Dentistry, Alexandria University, Champlion St., Azarita, Alexandria, Egypt; 2 Lecturer of Pharmacology and Experimental Therapeutics, Medical Research Institute, Alexandria University, Alexandria, Egypt; 3 Assistant professor of Oral Biology, Faculty of Dentistry, Alexandria University, Alexandria, Egypt

**Keywords:** Tongue, Muscles, Nerves, Electronic cigarettes, Vitamin C, Vitamin E

## Abstract

**Background:**

This study evaluated the ultrastructural effects of electronic cigarettes’ (EC) exposure in rat lingual nerves and muscles and assessed the therapeutic roles of vitamins C, E, and their combination.

**Methods:**

Forty adult male albino rats were allocated into 5 groups. Control: injected saline intraperitoneally, EC group: injected EC-liquid containing nicotine at a dose of 0.75 mg/kg, EC + C group: injected with EC-liquid and then supplemented orally with vitamin C, EC + C group injected with EC-liquid and then supplemented orally with vitamin E, and EC + C&E group: injected with EC-liquid and then supplemented orally with a combination of both vitamins. Transmission electron microscopy (TEM) and oxidative stress biomarkers malondialdehyde (MDA), and superoxide dismutase (SOD) were used to assess tissue damage and antioxidant effects.

**Results:**

EC group showed disrupted myelin sheaths, abnormal mitochondria, elevated MDA, and reduced SOD activity, indicating oxidative damage. EC + C group showed muscular recovery but did not significantly improve oxidative markers (*p* > 0.05) when compared to EC group. EC + E and EC + C&E groups showed regular myelin sheaths, normal mitochondria, and significant improvements in MDA and SOD levels compared to the EC group (*p* < 0.01).

**Conclusions:**

Vitamin E alone or combined with vitamin C effectively mitigates EC-induced oxidative stress and ultrastructural damage in muscular and nerve tissues. Vitamin C alone offers insufficient protection, mostly supporting muscular recovery without significantly improving nerve integrity or oxidative status.

## Introduction

The use of electronic cigarettes (ECs) has become a preferred replacement method for nicotine in smokers. The striking increase in the consumption of ECs is due to various ranges of appealing flavors, in addition to the concept of being a less harmful alternative to conventional cigarettes [37]. The main components of electronic cigarette (EC) liquid solutions are propylene glycol, vegetable glycerin, flavors, and nicotine. [15, 34].

The exact mechanism of EC toxicity remains unclear and requires further research. Studies have shown that the toxic effects of EC are associated with oxidative stress and inflammation [10]. Moreover, ECs are associated with altered central nerve activity and decreased muscle glycogen content. [43].

Oxidative stress occurs as a result of physiological imbalance between the levels of antioxidants and oxidants. Failure of the body’s natural antioxidant defense mechanisms to stabilize the excess production of free radicals results in a state of a cellular oxidative environment. [5, 9, 27]. Antioxidant defense mechanisms can be classified into two categories: endogenous antioxidant enzymes that are generated by the body itself, such as catalase, superoxide dismutase (SOD), and glutathione peroxidase (GSH-Px), and exogenous nonenzymatic antioxidants that could be attained through food, such as ascorbic acid (vitamin C), α-tocopherol (vitamin E), and vitamin A [2, 26].

The peroxidation of lipids in the cell membrane during oxidative stress generates mutagenic end products, such as lipid hydroperoxides and malondialdehyde (MDA). Consequently, MDA levels are used as indicators of oxidative and cellular damage [52]. Mitochondria are highly dynamic cell organelles essentially involved in cellular metabolism and energy production [9]. The superoxide dismutase (SOD) enzyme is considered a mitochondria-specific antioxidant. During the electron transport chain, the SOD enzyme can detoxify oxygen ions by the formation of hydrogen peroxide (H_2_O_2_) which is ultimately converted into water (H_2_O) [38].

Vitamins are inexpensive yet essential for overcoming oxidative stress. Vitamin C is a water-soluble vitamin that is required for multiple metabolic processes. It protects the aqueous cellular portion, mainly the cytoplasm, from oxidative damage and free radicals [47, 58]. In addition to acting as a free-radical scavenger by electron donation, vitamin C modulates the expression of the procollagen gene and thus can impact fibroblast differentiation and collagen formation [41, 54]. Moreover, vitamin C serum levels are inversely associated with inflammatory markers [14]. A sizable percentage of our bodies is composed of skeletal muscles, where approximately 70% of the total amount of vitamin C is stored. Vitamin C acts as a reducing agent against the produced oxidative damage that induces endoplasmic reticulum stress and ROS production in skeletal muscle cells [47].

Vitamin E is a membrane-bound antioxidant that is considered the first protective barrier to the cells by protecting the cell membrane from lipid peroxidation. Vitamin E acts as a free radical scavenger via an electron transfer mechanism to unveil an oxidized vitamin E cation radical. [47, 50]. Due to the lipophilic antioxidant properties of vitamin E, it protects the lipid integrants of cellular membranes and maintains membrane fluidity [4, 5, 58].

Some vitamins can act synergistically. Vitamin C is capable of neutralizing radical forms of vitamin E, transforming them to their reduced form and regenerating their antioxidant effect. A combination of both vitamins C and E has protective effects on smoke-induced oxidative stress, which is due to their radical-scavenging ability [30, 41, 47].

Owing to the continuous state of oxidative stress and increased rate of turnover of antioxidants in smokers, studies designed to measure the serum levels of vitamins C and E have shown a significant decrease in the serum levels of both vitamins among smokers [16, 27]. The efficacy of vitamins E and C in reversing the cytotoxic effects of nicotine in human gingival fibroblast cell culture has shown promising results regarding cell morphology and viability [58].

The objective of the current study was to examine the deleterious effects of electronic cigarettes on the lingual muscles and nerves. In addition, this study aimed to examine the role of antioxidant vitamins in alleviating oxidative stress caused by electronic cigarettes. Moreover, we focused on comparing the effects of vitamins C and E alone with those of their combination. Although the use of antioxidant supplements has increased recently, their impact on our biological system, depending on their type and dosage, is still not fully understood to avoid their overuse.

## Materials and methods

### Ethical approval

The Research Ethics Committee, Faculty of Dentistry, Alexandria University approved the current study IRB: 0678–5/2023. The current experiment complied with the Helsinki Declaration, ARRIVE guidelines, and was be carried out in accordance with the U.K. Animals (Scientific Procedures) Act, 1986 and associated guidelines, EU Directive 2010/63/EU for animal experiments, and the National Research Council's Guide for the Care and Use of Laboratory Animals (NIH Publications No. 8023, revised 1978).

### Study subjects

In this study, forty adult male albino rats weighing (200–250 gms) were used in the experimental animal house of the Faculty of Medicine, Alexandria University. The number of animals in this study was estimated based on calculations of the sample size made in the Department of Biomedical Informatics and Medical Statistics, Medical Research Institute, Alexandria University. To fulfill the 80% power of the study and 95% confidence level, the minimum required sample size was found to be 8 rats per group (number of groups = 5) (total sample size 40 rats) based on previous literature reviews [1, 48].

The animals were housed in specially designed wire mesh bottom cages, with four rats per cage, at room temperature and maintained under a light–dark cycle (L:D 12:12 with lights on at 07:00 h). They were supplied with a regular diet and drank tap water throughout the experiment.

### Experimental groups

The animals were randomly allocated into 5 groups via computer random number generator software (Prism G. version 5.01. GraphPad Software Inc: San Diego, CA, USA 2007) and the rats were given numbers ranging from 1—40:

- Control group: 8 rats were injected daily with 500 μl of saline intraperitoneally (i.p.) for 5 weeks.
- Electronic cigarette (EC) group: 8 rats were injected daily with EC-liquid containing 0.75 mg/kg of nicotine for 5 weeks [17, 19, 21, 45].
- Electronic cigarette and vitamin C (EC + C) group: 8 rats were injected daily with 0.75 mg/kg nicotine in the EC-liquid for 1 week. On day 8, they were supplied with vitamin C (300 mg/kg) [31] (Chemical Industries Development, Giza, Egypt) by orogastric gavage, 1 h after EC-liquid injection (for 4 weeks). The vitamin C tablets were dissolved in drinking water.
- Electronic cigarette and vitamin E (EC + E) group: 8 rats were injected daily 0.75 mg/kg nicotine in the EC-liquid for 1 week. On day 8, they were supplied with vitamin E (60 mg/kg) [59] (Pharco Pharmaceuticals, Alexandria, Egypt) by orogastric gavage, 1 h after EC-liquid injection (for 4 weeks). The vitamin E capsules were cut open and dissolved in corn oil.
- Electronic cigarette and vitamins C and E (EC + C + E) group: 8 rats were injected daily with 0.75 mg/kg nicotine in the EC-liquid for 1 week. On day 8, they were given a combination of vitamin C (300 mg/kg) and vitamin E (60 mg/kg) by orogastric gavage, 1 h after EC-liquid injection (for 4 weeks). [31, 57, 59].

### Administration of electronic cigarette refill liquid

Commercially available electronic cigarette refill liquid was acquired. The electronic cigarette refill bottles purchased were of tobacco flavor, and the concentration of nicotine was 12 mg/ml. The EC refill liquid contained 40% propylene glycol and 60% vegetal glycerin. EC injections were (11.7 μl) dissolved in saline (total volume 500 μl/rat). The nicotine dose chosen (0.75 mg/kg) is equivalent to the daily nicotine intake in subjects who smoke 10–20 cigarettes per day [7, 45]. EC refill liquids were freshly prepared each day and administrated intraperitoneally once daily, always at the same time of the day for all the animals [7, 23, 35, 51].

### Tissue harvest

All animals were sacrificed 1 day after the last injection by cervical dislocation to retrieve their lingual tissues. The animals were placed under deep isoflurane anesthesia (4%) to ensure the well-fare and painless euthanization of all animals. Cervical dislocation was performed by well-trained veterinary staff in the animal house located in the Faculty of Medicine, Alexandria University. The method used complied with the American Veterinary Medical Association (AVMA) Guidelines for the Euthanasia of Animals (2020 Edition) [29].

### Blood analysis

Blood samples were collected from the abdominal aorta of euthanized rats into plain vacuum tubes and allowed to clot at room temperature for the detection of (MDA), and (SOD). Blood samples were centrifuged for 10 min at 6000 rpm.

The MDA colorimetric assay was carried out as reported by Ohkawa, H. Ohishi W, and Yagi K. where MDA reacts with thiobarbituric acid in the acidic medium at 95 °C to form the thiobarbituric acid reactive pink color product. The absorbance of this product was measured at 534 nm and was positively correlated with the MDA concentration [44].

SOD activity was detected via a colorimetric method based on the Nishikimi method which relies on the ability of SOD to inhibit the phenazine methosulfate (PMS)-mediated reduction of nitroblue tetrazolium dye. The percentage inhibition was measured by comparing the change in the absorption at 560 nm for 5 min between the serum samples and the control sample following the addition of PMS [40].

### Histological analysis

For light microscopic examination, the specimens were fixed in 10% neutral buffered formalin for 48 h, dehydrated in ascending concentrations of ethanol, infiltrated then embedded in paraffin wax. Sagittal 4 μm thick sections were stained with hematoxylin and eosin (H&E) for histological evaluation.

### Ultrastructural examination using transmission electron microscopy (TEM)

The ventral segments were cut into small pieces, fixed in 2.5% glutaraldehyde for 24 h, and then they were washed with phosphate buffer (0.1 M, pH 7.4). Following the routine steps of TEM, the sections were examined, and photographs were taken via a JEOL transmission electron microscope (Japan). The examination was performed in the Faculty of Science, Alexandria University.

### Statistical analysis

The Kolmogorov-Smirnov test of normality revealed no significant differences in the distribution of the variables, so the parametric statistics were adopted. The data are presented as the minimum, maximum, mean, standard deviation, standard error of the mean, 95% CI of the mean, median, and 25^th^−75^th^ percentile (interquartile range) [22]. Comparisons were carried out between more than two independent normally distributed subgroups using one-way analysis of variance (ANOVA) test [39]. When the F ratio of ANOVA was significant Levene test of homogeneity of variances was done, and if significant Brown-Forsythe Robust test was adopted. Post-hoc multiple comparisons were performed using Games-Howell test.

## Results

### Light microscopic examination

**Fig. 1:**
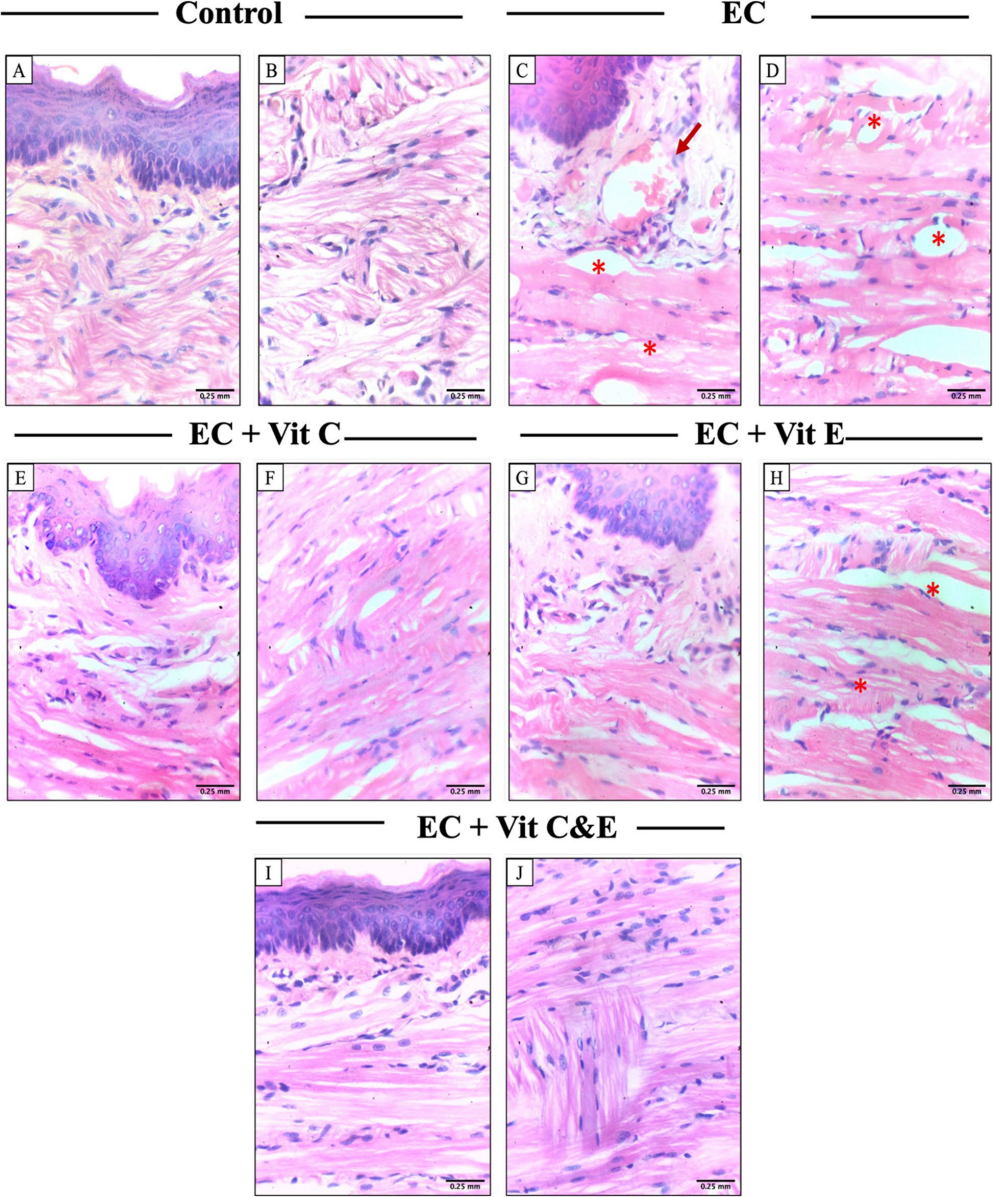
effect of electronic cigarettes with and without vitamin C & E supplementation using light micrographs, H & E stain. X400, control: (**A**, **B**) regular arrangement of muscular bundles. EC: (**C**, **D**) Vacuolation areas (asterisk), blood vessel (arrow). EC + C: (**E**, **F**) normal muscle organization. EC + E: (**G**, **H**) Vacuolation areas (asterisk). EC + C & E (**I**, **J**): regular arrangement of muscular bundles

The control group showed a regular arrangement of muscular bundles, with proper integrity of muscle fibers, as shown in Fig. (1A, B). The EC group exhibited myodegeneration and dystrophy. Vacuolation areas appeared between muscle bundles. (Fig. 1C, D). The EC + vit C group showed a degree of muscular regeneration and regular arrangement of muscular bundles with dense muscle fibers (Fig. 1E, F).

On the other hand, the EC + vitamin E group showed less muscular organization than did the group supplemented with vitamin C. Vacuolation and myodegeneration were observed among muscle bundles (Fig. 1G, H). EC + vit C & E showed the most comparable results to those of the control group. The muscular restoration was evident. A regular arrangement of muscular bundles with dense muscle fibers was observed (Fig. 1I, J).

## Transmission electron microscopy (TEM) examination

### TEM examination of the lingual muscles

**Fig. 2:**
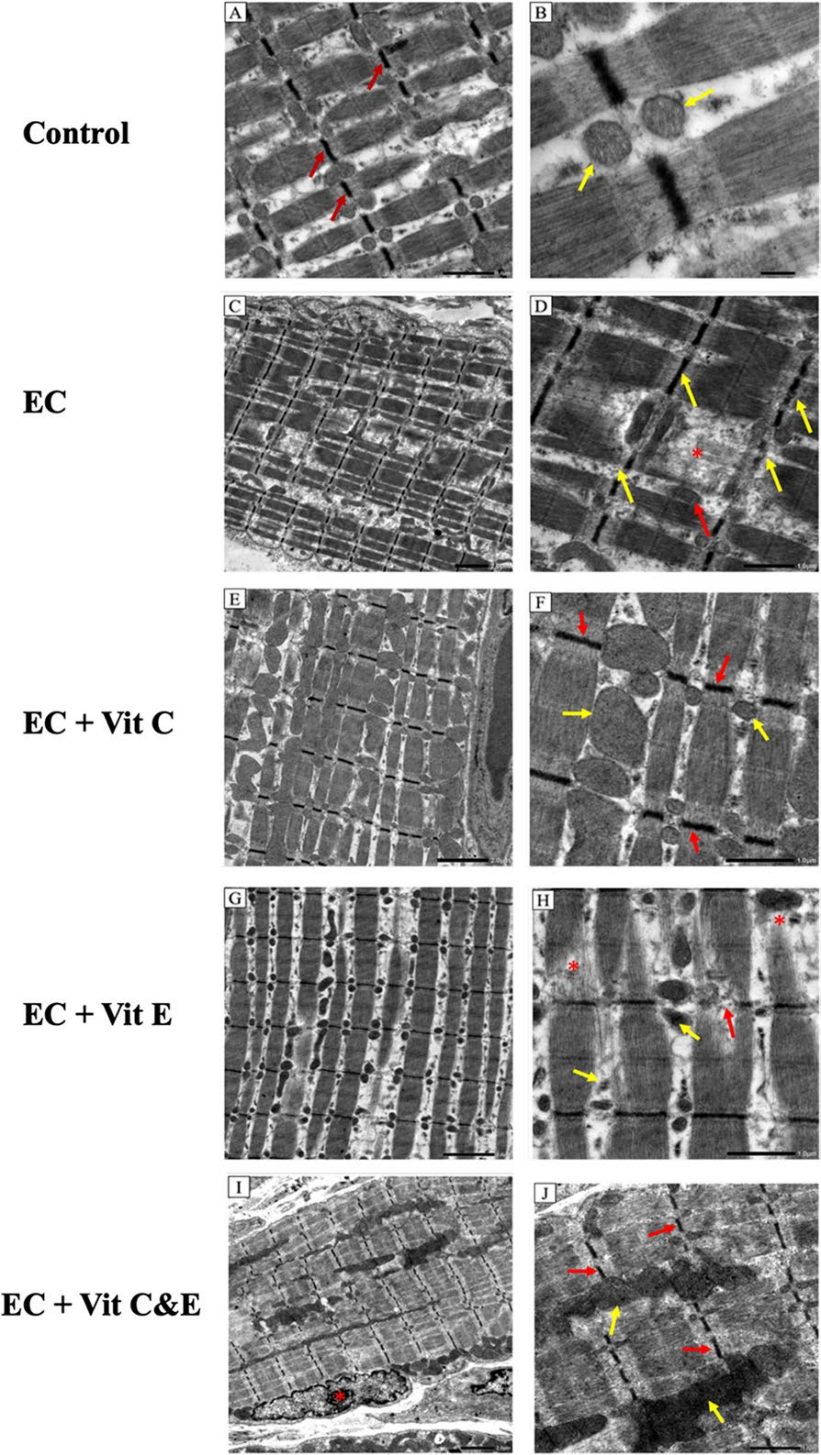
The effect of electronic cigarettes with and without vitamin C & E supplementation on lingual muscles using TEM, Control (**A**, **B**): (2A) dense z lines (red arrow), (2B) normal mitochondria (yellow arrows). EC [C, D]: (2C) myofibrillar derangement. (2D) abnormal mitochondria (yellow arrow), interrupted z line (red arrow), degeneration of sarcomere (astrisk). EC + C (**E**, **F**): (2E) uniformly organized sarcomeres. (2F) abnormal appearance of mitochondria (yellow arrow), regularly organized z lines (red arrow). EC + E (**G**, **H**): (2G) degeneration of sarcomeres (2H): areas of myofibrils destruction (asterisk), abnormal appearance of mitochondria (yellow arrows), loss of z line (red arrow). EC + C & E (**I**, **J**): (2I) normal sarcoma arrangement and myocyte (asterisk). (2J) mitochondria regain normal appearance (yellow arrows), normal z line (red arrows)

Examination of the control group revealed a uniform arrangement of myofibrils in longitudinal arrays of sarcomeres bounded by regular Z lines. The normal appearance of the mitochondria was also noted in Fig. (2A, B). The EC group revealed a marked myofibrillar derangement, thinning, and destruction along with the abnormal appearance of mitochondria. In addition, interruption or complete loss of the z line was detected in Figs (2C, D). Examination of the electronic cigarettes and vitamin C (EC + C) group revealed evident muscle tissue recovery in the form of well-organized sarcomeres. Regular z line and mitochondria were regained in Fig. (2E, F). (Fig. 2).

The electronic cigarettes and vitamin E (EC + E) group exhibited a loss of normal muscular arrangement manifested by the destruction of myofibrils and discontinuity of the z line. The abnormal appearance of some of the mitochondria was also noted in Fig. (2G, H). The electronic cigarettes and vitamins C & E (EC + C & E) group revealed a typical organization of sarcomeres, a uniform z line, and normal mitochondrial appearance (Fig. 2I, J).

### Transmission electron microscopy (TEM) examination of the lingual neural elements

**Fig. 3:**
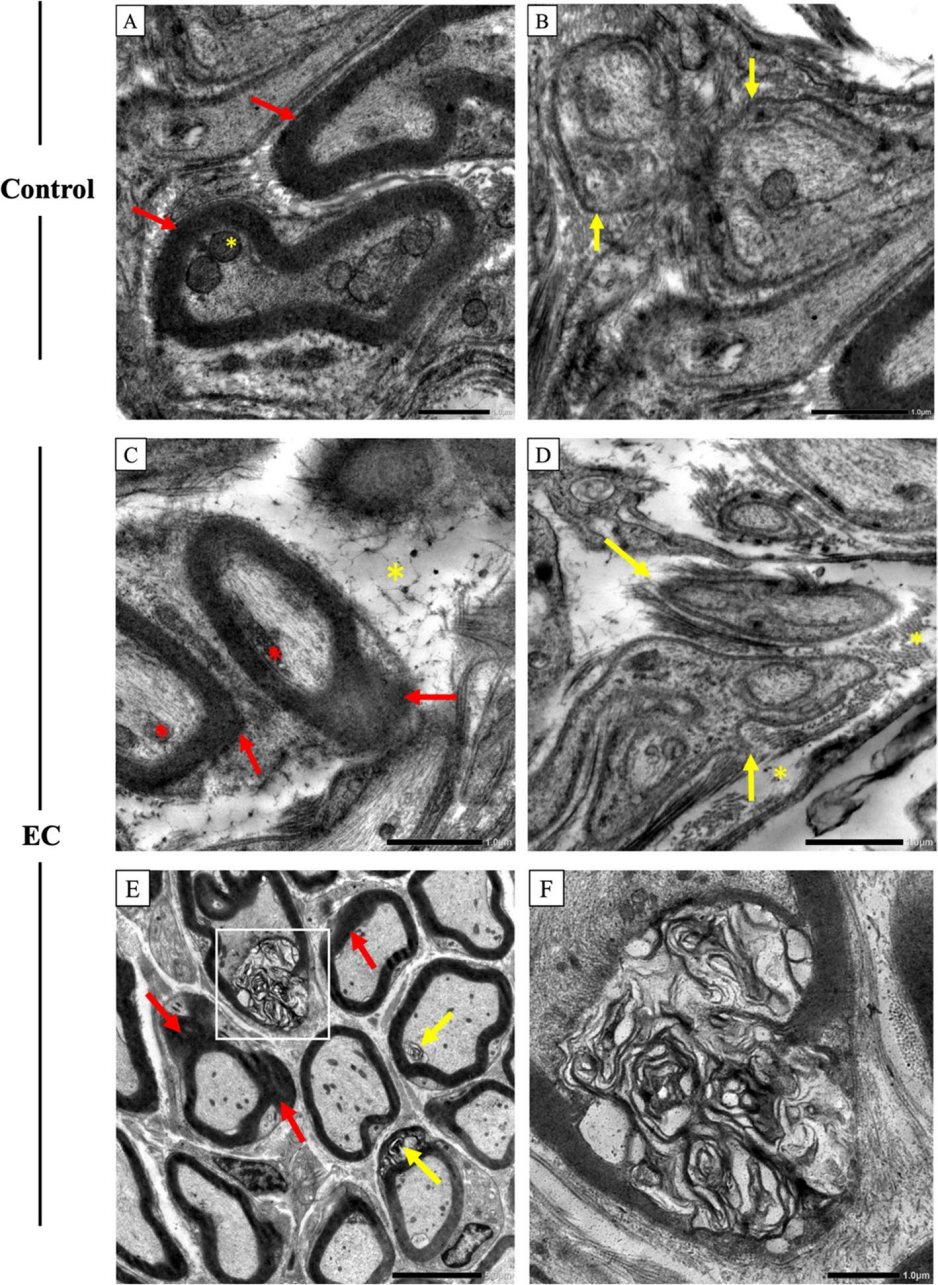
The effect of electronic cigarettes on lingual neural elements using TEM, Control (**A**, **B**): (3A) normal appearance of the myelin sheath (red arrow), normal mitochondria (asterisk). (3B) nonmyelinated nerve (yellow arrow). EC (**C**−**F**): (3C) myelin sheath out folding (red arrow) (3D) unmyelinated nerve fibers enveloped by disorganized thin lamina of Schwann cell sheets (yellow arrow). collagen fibers degeneration (yellow asterisk). (3E) myelin sheath out folding (red arrow), myelin figures (yellow arrow). (3F) magnified inset shows myelin figures

Examination of the control group revealed myelinated nerve fibers with dense myelin sheaths and normal mitochondria and neurofilaments in the axoplasm, as shown in Fig. (3A). Normal unmyelinated nerve fibers containing mitochondria and regular microtubules, appeared. Dense collagen fibers were also noted around the nerve in Fig. (3B). On the other hand, examination of the EC group revealed different forms of myelin sheath degenerative changes, including areas of thickening, and outfolding. Split myelin sheath contained variable cytoplasmic vacuoles and whorled myelin figures. Unmyelinated nerve fibers were enveloped by disorganized, thin laminae of Schwann cell sheets. The endoneurium showed obvious collagen fiber degeneration Fig. (3C, D, E, F). (Fig. 3).

**Fig. 4:**
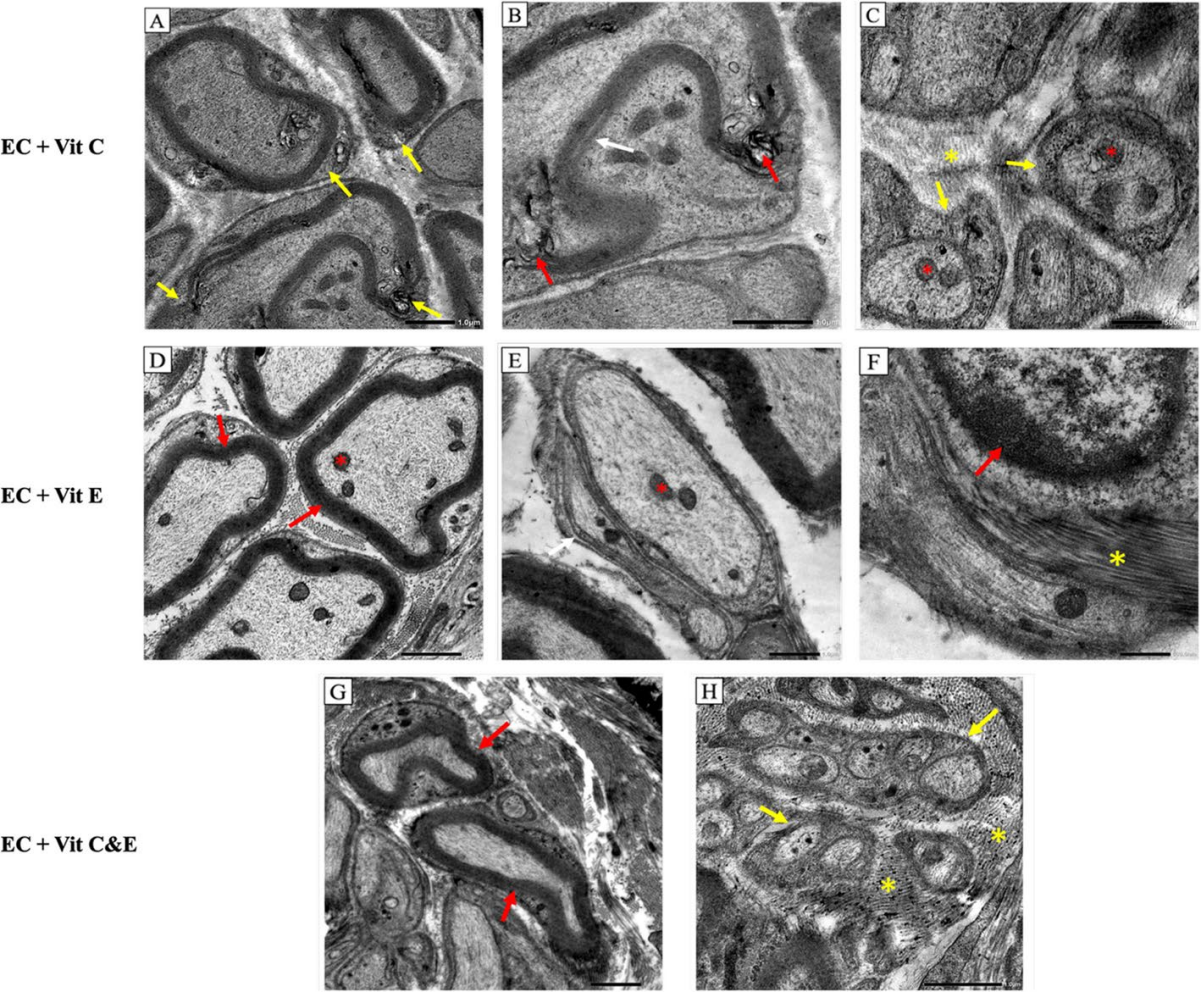
The effect of electronic cigarettes with vitamin C & E supplementation on lingual neural elements using TEM, TEM, EC + C (**A**-**C**): (4A) abnormal appearance of the myelin sheath (yellow arrow), (4B) higher magnification shows interrupted myelin sheath with myelin figures (red arrow), splitting of the myelin sheath (white arrow). (4C) Schwann cell membrane (yellow arrow), normal mitochondria (red asterisk), uniform collagen bundles (yellow asterisk). EC + E (D-F): (4D) normal appearance of myelin sheath (red arrow), normal mitochondria (red asterisk), (4E) unmyelinated nerve shows intact mitochondria (red asterisk), (4f) dense uniform collagen bundle (yellow asterisk), Schwann cell nucleus (red arrow). EC + VIT C & E (G, H): (4G) normal organization of myelinated axons surrounded by high density of collagen fibers (4H) unmyelinated nerve fibers enveloped by thin lamina of Schwann cell sheets (yellow arrow). Nerve fibers bundle surrounded by collagen fibers (yellow asterisk)

The electronic cigarettes and vitamin C (EC + C) group showed nerve alterations in the form of myelin sheath destruction and splitting. Myelin figures were evident signs of degeneration in Fig. (4A, B). Unmyelinated nerve fibers were enveloped by Schwann cell sheets. However, normal mitochondria and uniformly arranged collagen fibers were detected in Fig. (4C). Meanwhile, the electronic cigarettes and vitamin E **(**EC + E) group revealed myelinated nerve fibers enveloped by a heavy uniform myelin sheath with axoplasm showing regular regularly organized neurofilaments and normal mitochondria (4D). Unmyelinated nerves showed intact mitochondria and were surrounded by dense uniform collagen bundles in Fig. (4E, F). Electronic cigarettes and vitamin C&E (EC + C & E) group revealed a thick uniform myelin sheath ensheathing the myelinated nerve fibers, also normal mitochondria were noted. (Fig. 4G). Unmyelinated nerve fibers showed typical uniform ultrastructure and arrangement. Dense regular collagen fibers of endoneurium were restored (Fig. 4H).

### Blood analysis

The current study explored the effects of electronic cigarettes (EC), electronic cigarettes and vitamin C (EC + C), electronic cigarettes and vitamin E (EC + E), and electronic cigarette and vitamins C and E group (EC + C&E) on the serum level of the lipid peroxidation product malondialdehyde (MDA, mmol/ml) and on the activity of superoxide dismutase (SOD) enzyme (U/ml). The results revealed that EC significantly increased MDA level compared with that in the control group (*p* = 0.001), with a mean ± standard deviation (SD) values of 4.74 ± 0.75 and 20.66 ± 6.03 for the control and EC groups, respectively.

The addition of vitamin C alone to EC-exposed rats resulted in a nonsignificant decrease in MDA level compared with that in the EC group (*p* > 0.05) with a mean ± SD of 16.15 ± 4.84. On the other hand, treatment of EC-exposed rats with vitamin E alone or in combination with vitamin C significantly decreased the MDA level compared with that in the EC group, with a mean ± SD of 10.40 ± 2.10 for the (EC + E) group and 12.01 ± 2.96 for the (EC + C & E) group (*p* < 0.05). In addition, the MDA levels in all the vitamin-treated groups ((EC + C), (EC + E) and (EC + C&E) were significantly greater than those in the control group.

Parallel results were obtained for superoxide dismutase (SOD) activity, where both EC and (EC + C), produced a statistically significant decrease in SOD activity compared with that in the control group (*p* < 0.001), with no significant difference between the EC and (EC + C) groups (*p* > 0.05). The mean values ± SD for the control, EC and (EC + C), groups were 65.38 ± 6.95, 32.50 ± 6.39 and 34.87 ± 5.84 respectively. However, (EC + E), and (EC + C&E) groups presented significantly greater SOD activity than did the EC group, as well as the (EC + C) group (*p* < 0.01) with mean ± SD, was 45.88 ± 5.91 for (EC + E) and 50.50 ± 8.35 for (EC + C&E) group. However, these achieved increases in SOD levels were still significantly lower than those in the control group (*p* < 0.05) (Table 1).

## Discussion

The safety of electronic cigarettes (ECs) is debatable, considering the current research, which has demonstrated that the use of ECs negatively affects oral homeostasis. Oxidative stress and elevated proinflammatory cytokine concentrations are associated with their cytotoxic effects [13].

The effects of smoking on the muscles and nerve fibers of the tongue have not been sufficiently studied. Therefore, the current study focused on ultrastructural changes induced by ECs in muscle and neuronal components of the ventral tongue surface. In addition, we investigated the alleviating effect of ECs administered with vitamin C and vitamin E alone vs a combination of both vitamins. Vitamin C plays an essential role in the antioxidant effect of vitamin E. It converts the oxidized forms of α -tocopherol back to α-tocopherol and thereby reactivates vitamin E [36].

The intraperitoneal injection method was adopted in the current study because it is easy, reliable, and allows the delivery of liquid directly to the kidneys, eliminating other pathways of metabolization and excluding the effects of heat produced by smoking [17, 21, 35]. Instead, we focused only on the direct effects of EC constituents on lingual muscles and nerves [7, 35, 51]. Nogueira et al. measured plasma cotinine levels and reported that cigarette smoke components whether delivered by inhalation or into the circulation by intraperitoneal injections negatively impact skeletal muscles [42].

Moreover, a study compared between effects of EC-liquid with versus without nicotine in rat epididymis. Both groups showed negative histological effects and a significant elevation in MDA was observed in comparison to control group. These findings were attributed to propylene glycol exposure or flavoring agents present in EC liquid [49]. El Golli et al. concluded that nicotine alone caused fewer histopathological changes when compared to EC-liquids containing nicotine [17, 21]. Furthermore, several in vitro studies reported a cytotoxic effect of not only the aerosol but also EC-liquid on various cell cultures. It was concluded that the cytotoxicity of EC liquid may be related to its high concentrations of flavoring chemicals [8, 25, 33]. Accordingly, the negative effects of electronic cigarettes are not only confined to the presence of nicotine but also to additional components such as propylene glycol or flavoring agents and could be attributed to the direct toxic effect of the EC liquid.

In the current study, the EC group revealed a marked decrease in muscular density with evident areas of myofibrillar degeneration and abnormal mitochondrial appearance. These findings are in accordance with those of Chen et al. [11]. On the other hand, the EC group supplemented with vitamin C showed muscular recovery. Moreover, the EC group supplemented with both vitamins C and E exhibited typical organization of sarcomeres, a uniform z line, and normal mitochondria.

Although nicotine may increase muscle blood flow [11], it negatively affects the structural architecture of muscular fibers, as it leads to mitochondrial dysfunction,thus, it negatively impacts skeletal muscle structure and function. [42]. The high affinity of nicotine for mitochondrial neuronal nicotinic acetylcholine receptors (nAChRs) is connected to apoptotic changes and has a negative impact on mitochondrial metabolism [32, 60]. It directly affects mitochondrial respiration, cell autophagy, and cell signaling molecules. Moreover, the altered mitochondrial dynamics and mitochondrial membrane potential cause mitochondrial hyperfusion and swelling [53, 61].

Espinoza-Derout et al. and Hasan et al. evaluated the effect of ECs on cardiac muscles. TEM examination revealed derangement, thinning, and destruction of cardiac myofibrils [20, 24]. Chou et al. reported that short-term administration of vitamins C and E on exercise-induced tissue damage effectively reduced muscle damage and inflammatory responses [12]. Another study was conducted to evaluate the effect of vitamin C on cardiomyopathy. TEM examination revealed regeneration of myocardial fibers and distinct Z lines along with well-preserved mitochondrial integrity [56]. This finding is in agreement with the current study. However, more histological research needs to be done to compare vitamins C, E and their combination.

Ultrastructural examination of nerves in the current study revealed myelinated fibers with abnormal areas in the (EC) group. Moreover, some axons exhibited abnormal myelin figures in both EC and (EC + C). Myelin figures are composed of whorled phospholipid masses produced from the myelin sheath. This is a sign of cell injury and apoptosis. On the other hand, groups supplemented with vitamin E or a combination of C and E showed normal myelinated nerve fibers with even thickness of myelin sheath. Nonmyelinated nerves were surrounded by dense collagen fibers. These findings are in accordance with those of Sayed et al. and Elgayar et al. [18, 55]. The normality of neuronal mitochondrial function is crucial in the high energy demand seen in nerves. Vitamin E has the potential to inhibit lipid peroxidation in the brain and acts as a neuroprotectant. [6].

In 2023, a study evaluated the protective effects of vitamins C and E against deleterious impact of EC liquid on lingual papillae. Supplementation with a combination of both vitamins resulted in remarkable preservation of the normal histological features of the lingual papillae and mucous membrane, indicating the remarkable synergic effect of both vitamins [57].

This study compared the effects of vitamin C and E supplementation with supplementation of only one of these vitamins on the ultrastructural changes caused by electronic cigarette liquid in rat tongue nerves and muscles. Recently, there has been overconsumption of vitamins, which can have serious implications on human health. Thus, this study design has clinical relevance in confirming the beneficial use of combined vitamins and ensuring that vitamins are not overused.

The results of the present study revealed that, compared with the control group, the EC group presented a statistically significant increase in malondialdehyde (MDA) levels and a decrease in superoxide dismutase (SOD) activity. The addition of vitamin C resulted in a nonsignificant decrease and increase in MDA level and SOD activity respectively compared with those in the EC group. However, compared with those in the EC group, the rats treated with vitamin E alone or in combination with vitamin C presented a statistically significant decrease in the MDA level and a statistically significant increase in SOD activity.

Similarly, in 2021, an in vivo study was carried out to study the effect of ECs on the colonic mucosa. In the exposed group, the MDA levels increased significantly, whereas the SOD levels decreased [37]. Moreover, Paunović et al. reported significantly lower SOD levels in albino rats that were intraperitoneally injected with nicotine (0.75 mg/kg/day) than in the control group [45].

In addition, a 2020 clinical study was conducted to compare the level of oxidative stress between smokers and nonsmokers. This study reported that the total reactive oxygen species (ROS) content was significantly greater in both tobacco smokers and EC smokers than in nonsmokers, with no significant difference between the two types of smokers [28]. Nicotine itself or its active metabolite, cotinine, can increase MDA levels and decrease SOD activity through the enhancement of lipid peroxidation by inducing the cytochrome P450 enzyme system, nicotinamide adenine dinucleotide phosphate (NADPH) oxidase activity and myeloperoxidase activity [31]. In addition, Pinkston et al. reported that nicotine and flavored aerosols in ECs have immunosuppressive effects as a result of oxidative stress induction [46].

In contrast to our results regarding vitamin C, Ahmed et al. reported that, compared with that in nicotine-treated rats, SOD activity in the splenic tissues of rats was significantly greater after treatment with vitamin C before subcutaneous nicotine injection [3]. However, the SOD level was significantly lower than that in the control group. This dissimilarity could be explained by that Ahmed et al. started the treatment with vitamin C simultaneously with nicotine administration. However, in the present study, vitamin C administration was started after 1 week of administration of EC refill liquid to achieve nicotine dependence. Therefore, the increase in SOD activity and decrease in MDA level achieved in our study could reach a significant value if treatment with vitamin C started earlier.

Similarly, a longer treatment interval significantly decreased the MDA levels in the hepatic and renal tissues of rats treated with vitamin C + nicotine compared with those treated with nicotine alone. In this study, 300 mg/kg vitamin C was injected intraperitoneally 3 days before intraperitoneal nicotine injection, throughout nicotine administration (3 weeks), and 2 days after nicotine injection [31]. Furthermore, the intraperitoneal route of administration used in the mentioned study to supplement vitamin C could confer a higher bioavailability than the oral route used in the current study.

Our results regarding the effect of vitamin E were consistent with the results of an in vivo study conducted in 2021. This study reported that 30 mg/kg vitamin E successfully increased the MDA level to a significant value compared with that in untreated cigarette smoke-exposed rats [5]. The ability of vitamin E to increase SOD activity and reduce MDA levels could be explained by its lipid solubility. This enables it to guard against lipid membrane peroxidation by scavenging ROS that attack the highly polyunsaturated fatty acid contents present in cell membranes. Thus, the different forms of vitamin E potentially exert antioxidant effects by disrupting the lipid peroxidation chain reaction [5].

Although vitamin E alone or in combination with vitamin C significantly improved oxidative stress markers compared with those in EC rats, they failed to reach the normal values for the MDA level and SOD activity observed in control rats. This finding reflects the damaging effects of ECs and nicotine even after the administration of antioxidants.

## Conclusion

EC had deleterious effects on both the lingual muscles and neural elements as well as the generation of a state of systemic oxidative stress, whereas supplementation with vitamins C and E combined alleviated these effects. Vitamin E, or a combination of C and E, mitigated EC-induced oxidative stress and ultrastructural changes affecting nerve fibers. Although vitamin C remarkably restored the normal structural architecture of muscle fibers, its supplementation alone was insufficient to reverse the oxidative damage affecting nerves.

## Study limitations

One limitation of our study that must be considered is that the intraperitoneal injection method of administration was chosen rather than inhalation. In fact, owing to the difficulty in establishing a system in which EC liquid is inhaled by rats, we used the i.p. route, which is largely used to assess nicotine toxicity in rodents. The intraperitoneal injection method allows the exclusion of heat during delivery. Thus, the findings of the current study need to be confirmed by aerosol experiments, to be applicable to humans. Moreover, this study design used only one type of EC liquid. We recommend comparing other types of EC liquid with different nicotine concentrations and flavors. Further studies should be conducted to evaluate the effects of EC devoid of any flavoring agents. Studying the possible protective and alleviating effects of vitamin C with longer treatment intervals is recommended. Further studies can be conducted to assess epithelial and inflammatory changes in EC-treated animals. Future studies could include a dose–response analysis of different concentrations of vitamins C and E to evaluate their protective effects against EC-induced damage.

## Data Availability

No datasets were generated or analysed during the current study.

## Appendix

Electronic cigarettes (EC).

Transmission electron microscopic (TEM).

Superoxide dismutase (SOD).

Malondialdehyde (MDA).

Glutathione peroxidase (GSH-Px).

Intraperitoneal (i.p.)

Phenazine methosulphate (PMS).

Hematoxylin and eosin (H&E).

Reactive oxygen species (ROS).

Nicotinamide adenine dinucleotide phosphate (NADPH).

Tumor necrosis factor-alpha (TNF-α).
